# Supplementary material for: Far-red pentamethine cyanine dyes as fluorescent probes for the detection of serum albumins
Source: R Soc Open Sci. 2020 Jul 1;7(7):200453. doi: 10.1098/rsos.200453 (PMC7428273; doi:10.1098/rsos.200453)
Supplement: Kovalska_figures_ESM [file rsos200453supp1.pdf]

Supplementary information

For

Far-red pentamethine cyanine dyes as fluorescent probes for the detection of serum albumins

D. Aristova[1], G. Volynets[1], S. Chernii[1], M. Losytskyy[1], A. Balanda[1], Yu. Slominski[2], A. Mokhir[3], S. Yarmoluk[1], V. Kovalska[1,4]

[1]*Institute of Molecular Biology and Genetics NASU, 150 Zabolotnogo St., 03143 Kyiv, Ukraine*

[2]*Institute of Organic Chemistry NASU, 5 Murmans'ka St., 02094 Kyiv, Ukraine*

[3]*Organic Chemistry II, Friedrich-Alexander-University of Erlangen-Nuremberg, Nikolaus-Fiebiger-Str. 10, 91058 Erlangen, Germany*

[4]*Scientific Services Company Otava Ltd, 150 Zabolotnogo St., 03143 Kyiv, Ukraine*

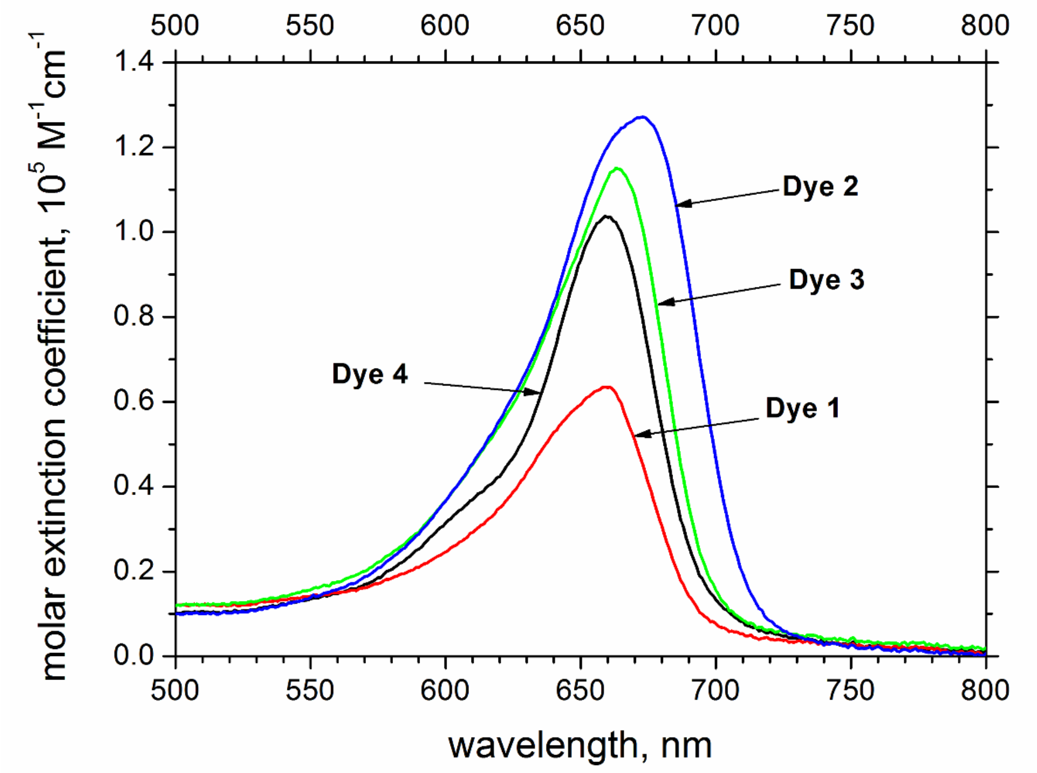

Figure S1 a. The absorption spectra of the studied dyes (1μM) in acetonitrile.

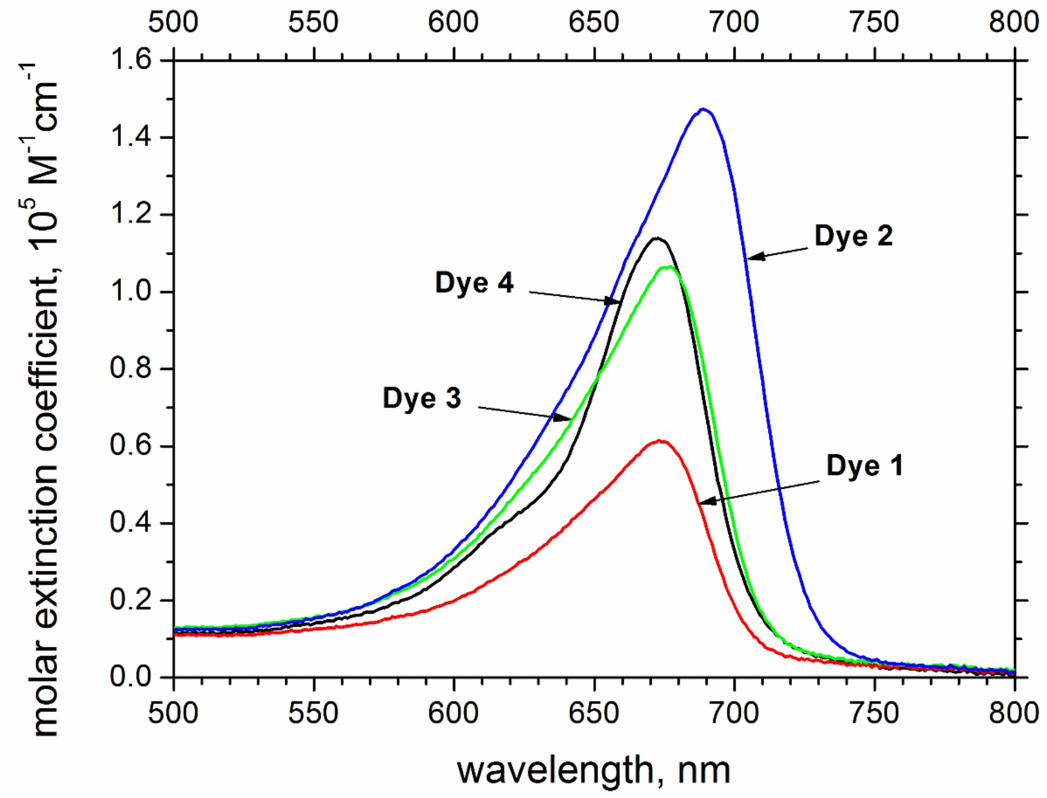

Figure S1 b. The absorption spectra of the studied dyes (1μM) in DMSO.

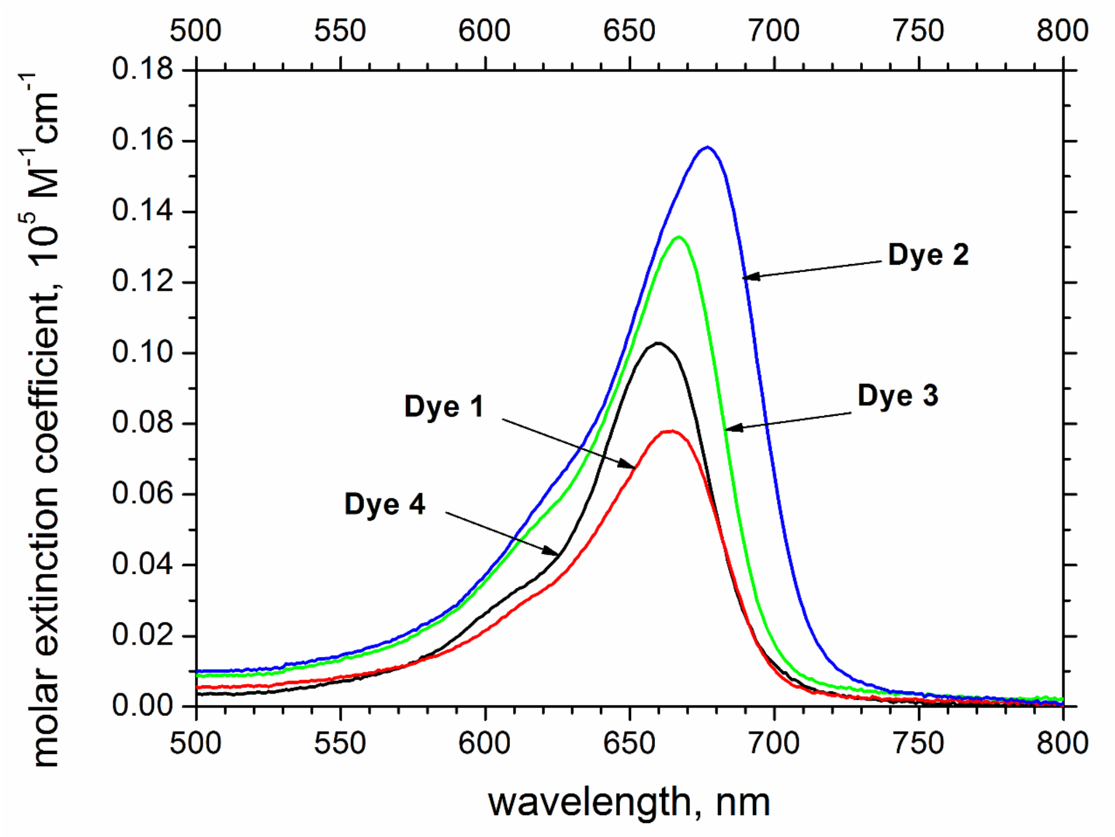

Figure S1 c. The absorption spectra of the studied dyes (1μM) in MeOH.

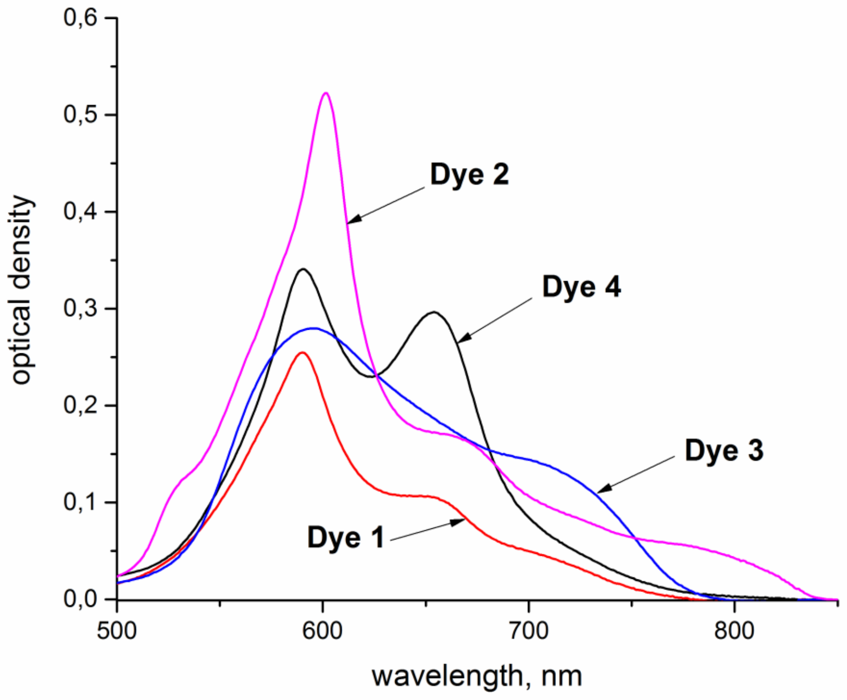

Figure S2 a. The absorption spectra of the studied cyanine dyes (10µM) in 0.05 M phosphate buffer (pH 6.0).

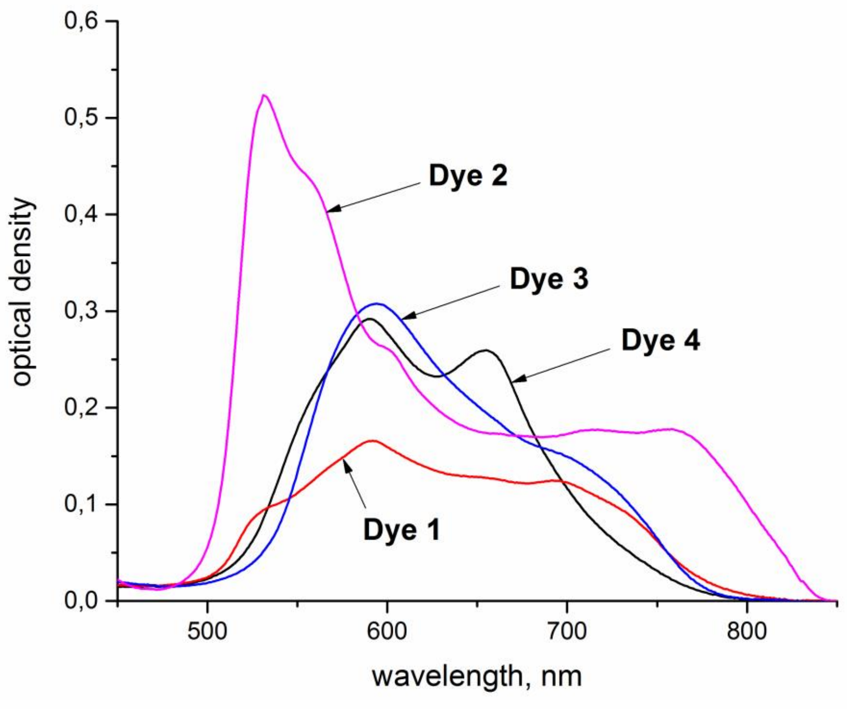

Figure S2 b. The absorption spectra of the studied cyanine dyes (10µM) in 0.05 M Tris-HCl buffer (pH 9.0).
